# Systematic Review of the profile of emergency contraception users

**DOI:** 10.1590/1518-8345.0882.2733

**Published:** 2016-07-04

**Authors:** Maria de Lluc Bauzà Amengual, Magdalena Esteva Canto, Inmaculada Pereiro Berenguer, Maria Ingla Pol

**Affiliations:** 1 MSc, Full Professor, Clinical Assistant Professor, School of Nursing and Physiotherapy, University of Balearic Islands, Mallorca, Spain.; 2 PhD, Researcher, Research Unit, Majorca Primary Care Department, Balearic Health Service, Majorca, Spain.; 3 Doctor, Health center primary care Puerto de Sagunto II, Valencia, Spain.; 4 Nurse, Health and consumer care department, Palma City Council, Mallorca, Spain.

**Keywords:** Contraception, Postcoital, Data Collection, Levonorgestrel

## Abstract

**Objective:**

to discern the profile of the Spanish Emergency Contraceptive users (EC). Design: systematic review of contraceptive use in the Spanish population. Data Source: Spanish and international databases, between January 2006 - March 2011.

**Keywords:**

Contraceptives, Postcoital pills, emergency contraception, levonorgestrel, data collection. Study selection: original papers, letters to the editor in which stated aims were the description, prediction or measurement of variables related to EC use. Twenty-two papers were retrieved and fourteen were finally selected, all of which were descriptive. Data extraction: manuscripts were evaluated by two independent reviewers.

**Results:**

Women requesting EC have ages between 21-24 years, mostly single and university students; declare that they have not previously used EC, and attend an Emergency department, at weekends and within 48 hours following unprotected sexual intercourse. The reason is condom rupture. None of the studies reviewed measured alcohol and other drug consumption, the number of sexual partners, nor any of the studies performed a comparison with a group not using EC.

**Conclusions:**

lack of homogeneity and comprehensiveness of studied variables resulted in a limited profile of Spanish EC users. Further studies are needed with a more comprehensive approach if sexual health interventions are to be carried out in possible users.

## Introduction

Throughout the world, unplanned pregnancy is a considerable social and public health issue. The majority of cases are unwanted pregnancies and many of these cases result in abortion. Pregnancies in girls between 14 and 17 years rose from 4‰ in 1990 to almost 12‰ in 2006^1^ This may have been due to two factors. Firstly, that contraception is either not used or is used incorrectly in many cases. Secondly, that penetrative sexual relations are being initiated at a younger age; and the younger age leads to a decrease in risk perception^2^

The rate of abortion or voluntary pregnancy termination (VPT) represents an estimate of the number of unwanted pregnancies. On analysing the incidence of this rate in Spain, we see moderate values with respect to other European countries ^3^. It should be pointed out that Spain is one of the countries that have seen the greatest increase in recourse to abortion in recent decades, rising from 7.14 VPT per 1,000 women in the year 2000, to 11.41 in 2009. The abortions are mainly in the 20-29 age group, and among those who have sophomore-10h-grade ^4^.

In the second half of the 20^th^ century, the emergence of contraception and its massive use in the developed world represented an effective measure for birth control and the avoidance of unwanted pregnancies. Lately, scientific advances have led to other types of drugs entering the market, known as emergency contraception (EC) or the 'post-coital pill'. EC use can be defined as taking a contraceptive drug (also known as the 'morning-after pill') up to 72 hours after unprotected coitus with the aim of preventing an unwanted pregnancy. Its mechanism of action is to impede ovulation or fertilisation, but once the fertilized egg is implanted the pill will not have any effect ^5^. The conclusions of clinical trials on the efficacy of these drugs support the use of Progestogen only (total dose of 1.5 mg of levonorgestrel) as the method of emergency contraception of choice due to greater effectiveness and lower incidence of nausea and vomiting ^6^. The introduction of these drugs to the market considerably raised expectations regarding prevention and reduction of the number of VPT and it has even been argued that its use could prevent up to 95% of unwanted pregnancies ^7^.

On March 23rd, 2001, the government in Spain authorised the sale of levonorgestrel as EC with a medical prescription. Since then, the various Spanish regions legalised its provision. This confirms that no common protocol exists in the National Health Service (NHS) establishing the conditions for the supply of EC among regional health services. The inclusion of EC in the catalogue of contraceptives has involved numerous debates and controversies. In 2003, 317,670 morning-after pills were dispensed, which corresponds to 3% of women of childbearing age, indicating a sharp increase in consumption ^8^. From September 2009, the so-called 'morning-after pill' could be acquired without a medical prescription in Spanish pharmacies. This measure aims to facilitate access to this pill for all women who require it, at the appropriate time to ensure its efficacy, irrespective of their place of residence and the regional laws in effect.

This deregulation of the pill and improvements in access to it, help to overcome certain obstacles for women such as the shame they may feel in consulting a health professional, as well as facilitating its anonymous purchase at pharmacies. From another point of view, such a high level of use could be interpreted as a failure, as access to contraception has not prevented the increase in abortions, nor has the availability of condoms reduced demands for the morning-after pill. Rather, it seems that these are factors which, taken together, may encourage people to enter into risky situations or remain in them ^9^. Thus, the improvements in EC accessibility have led to the formulation of three more pragmatic concerns ^10^: 1) Whether easy access to the EC pill increases early sexual activity, 2) Whether women using this method repeatedly tend to abandon their habitual contraceptives; and 3) Whether these factors expose women and their partners to a greater risk of sexually transmitted diseases.

Following these legislative changes, there is a need to analyse the current situation of women seeking EC in our country. Hence, the aim of this study is to discern the profile of EC users in Spain through a systematic review of the literature so that strategies can subsequently be designed to address the population of actual female users.

## Method

A systematic review of the literature published on emergency contraception use in the Spanish population was performed with the aim to discern the profile of the Spanish Emergency Contraceptive users. The bibliographic search was carried out in the main Spanish and international databases: PubMed, Cuidenplus, BDIE, CINAHL, EMBASE, Cochraneplus, ExcelenciaClínica, Joanna Briggs, IBECS, IME, OLID, ISOC and Ageline. The key words used were: Contraceptives, Postcoital pills, emergency contraception, levonorgestrel, data collection. Boolean operators and classical truncations were used (OR, AND, *).

Search inclusion criteria were as follows: original articles and letters to the editor published during the last five years (2006 to March, 2011) in English, Spanish or French, and directly or indirectly focused on the description, prediction or measurement of variables related to the use of emergency contraception in Spain.

A manual search was also performed using the references in the articles and reviews retrieved to identify those articles that had not been captured in the electronic search. Thus we were able to find articles published in Spanish journals that were not indexed in the above databases but had been subject of a peer-review process. Then, by reading the title and abstract, the papers meeting the inclusion criteria were selected. The full texts of the manuscripts selected were obtained so that pairs of researchers could subsequently assess them independently and analyse them through a grid designed for this study. To design the analytical grid, meetings were held within the research team until consensus was reached on the definitive analysis grid. Two reviewers retrieved data independently. Information was collected on the study design, aims, scope, participants, sample size, variables related to the use of EC, main results and conclusions. The variables gathered were socio-demographic variables, variables related to the use of EC (frequency, time elapsed since coitus, reason for use, prescription services) and variables connected with sexual habits (age at initiation of sexual relations, obstetrics history). The measures used are those presented in the studies, mainly percentages and means with standard deviations. The studies were evaluated separately and a general assessment was done to ensure they met minimum quality standards. The bibliographic reviews were conducted during the month of May of 2010 and during March of 2011.

## Results

**Figure 1:**
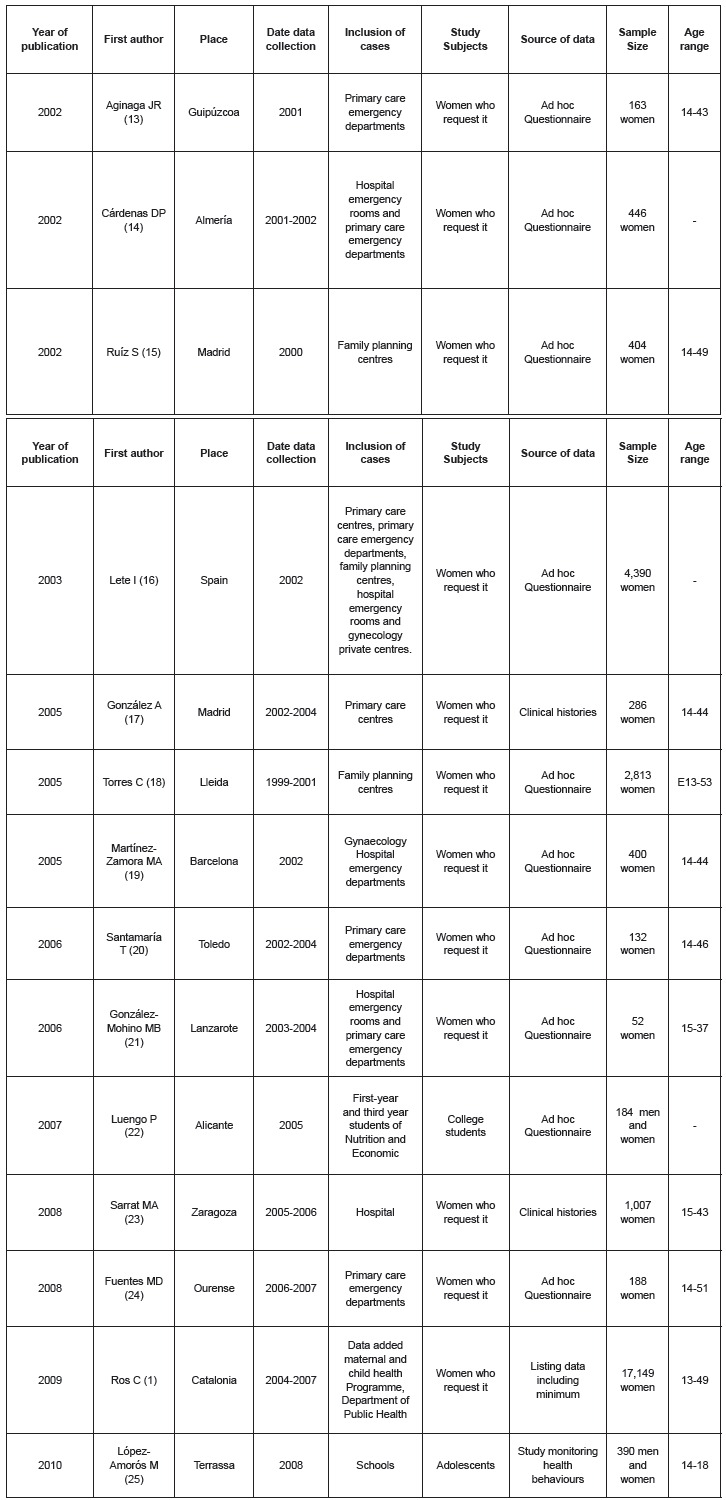
Selected articles on the use of the morning after pill in the Spanish population. Palma, Illes Balears, Spain, 2011

Twenty-two articles were retrieved and fourteen were finally selected, the majority (92.8%) in the Spanish language. Figure 1 shows the characteristics of the published articles ^11^ ^-^ ^24^. The studies selected were carried out between 1999 and 2008 and represent nine Spanish regions; all the studies had a descriptive cross-sectional design; no qualitative studies were found. In eight studies the data were collected from emergency services and the majority focused on women who requested EC, without a comparison group. Only two included both genders as the subjects of the studies dealt with adolescents and college students. Ages ranged from 13-53 years across the selected studies.

**Figure 2:**
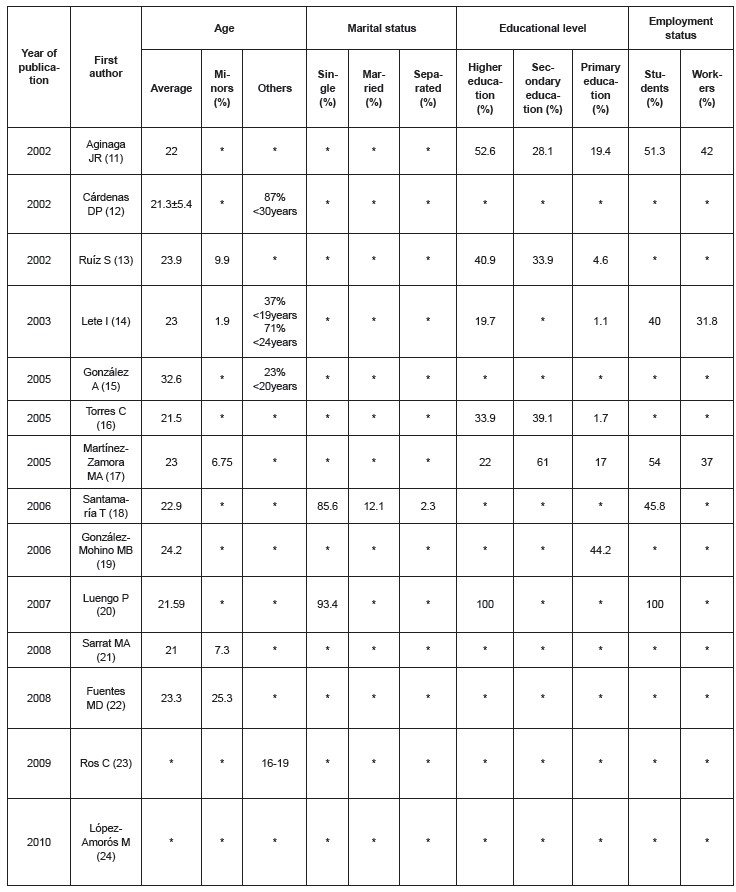
Distribution of socio-economic variables of women using emergency contraception, according to age, marital status, educational level and employment status. Palma, Illes Balears, Spain, 2011

Figure 2 shows the socio-demographic variables of the study subjects. In twelve of the fourteen articles the average age of the women who sought EC was reported and found to be between 21 and 24 years. In only half of the studies (7) information was provided on educational level showing that, among women requesting EC, university students accounted for between 19.7% and 52.6%. Only two studies collected data on civil status and these showed a high percentage of single women. Regarding employment, in the five studies that included this variable, the largest percentage of the study population was students.

*No data

**Figure 3:**
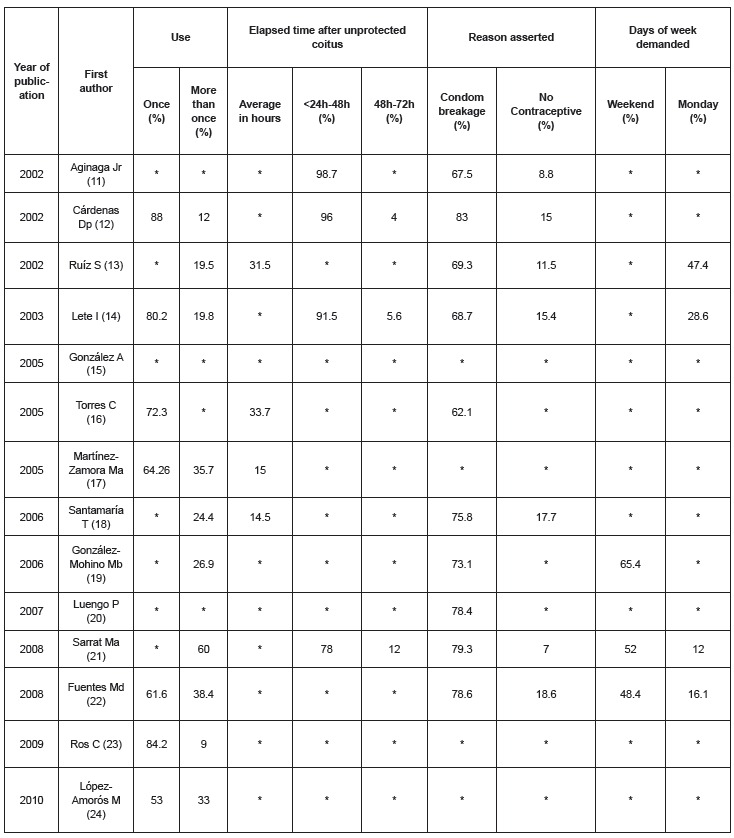
Variables distribution using EC, according to use, elapsed time after unprotected coitus, reason asserted and days of week demanded. Palma, Illes Balears, Spain, 2011

With respect to the use profile (Figure 3), the ten studies that included this variable reported that the majority of women stated that they had used EC only once. It should be noted that between 9-60% of women had used it on more than one occasion. In a study ^22^, 58.8% of repeated users were under 20 years of age. Time elapsed since unprotected coitus was recorded in eight studies and it appears that a very high percentage of women seek EC within 48 hours. The reason given by these women for requesting and using EC was reported in ten studies and mainly referred to condom breakage. In another study ^11^, 27.6% stated they used EC as a contraceptive method. Finally, the weekends were the time of greatest demand, followed by Mondays. It is noteworthy that some 7% of women interviewed in one study ^13^ continued to engage in unprotected coitus during the same menstrual cycle after taking EC.

*No data

**Figure 4:**
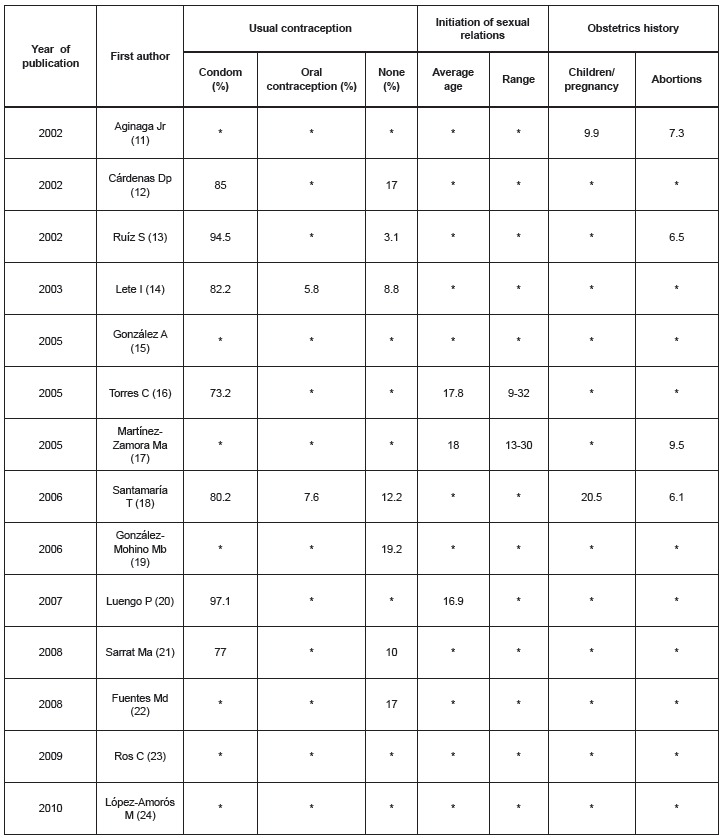
Sexual behaviour of women using emergency contraception, according to usual contraception, initiation of sexual relations and obstetrics history. Palma, Illes Balears, Spain, 2011

Sexual habits and habitual contraceptive method are shown in Figure 4. Data on the usual method of contraception were recorded in nine studies. All of these demonstrated that among EC users condoms were most frequently used, followed at some considerable distance, by oral contraception. Between 3% and 19% of the interviewees did not use any contraceptive method. Regarding the age at initiation of penetrative sexual relations, data were only gathered in three articles and was found to be between 16.9 and 18 years. Obstetrics history was recorded in few studies, reporting that between 6.1 and 9.5% of women surveyed stated having had an abortion on at least one occasion.

*No data

## Discussion

Based on these data, we discuss the effectiveness of the dispensation of this drug in Family Planning Centres as well as the inclusion of cases from these centres and those based on Clinical Histories.

There are Spanish regions where the prescription was free and others in which the drug was paid. As it was free in Andalucía, the article made in this region ^12^ should be analysed in a different manner.

Our study shows that, in general, the studies analysed present limited results that do not allow a clear, detailed picture to emerge of the characteristics of women using EC. Study results show great variability reflecting the various contexts in which the studies have been performed and cover primary care emergency departments, hospital emergency rooms, family planning centres, the general population, the university population, and secondary school students. This could explain, in turn, the considerable variability in the estimated parameters. The sample size in the published and selected studies used for analysis is also highly diverse, ranging from 52 up to 17,149 women. Moreover, it can be seen that the number of variables studied is limited and varies between studies, hampering attempts to view the profile of users in depth. For instance, studies performed outside Spain report on the use of other drugs among EC users and observe an association between consumption of alcohol and tobacco, number of sexual partners and use of EC, while in the reviewed studies these variables were not investigated ^25^ ^-^ ^28^. Nor was any study found where comparisons were made with women who had not taken EC, which would have allowed clarification of the characteristics that differentiate these women and the factors related to the use of EC. Similarly, in the United States and the United Kingdom, some qualitative studies have been carried out which provide an insight into the discourse of women on the free dispensation of EC in pharmacies or advance supply of 5 units of the drug ^29^ ^-^ ^32^. This research suggests that free dispensation increases access to EC that is highly valued by the women as it reduces waiting times , thus increasing efficacy as the drug is always administered within 72 hours of unprotected coitus. However, we did not find any qualitative studies performed in Spain, which further hinders the collection of detailed information on the views and experiences of women who use or are thinking of using EC. The availability of this type of study would also allow identification of relevant variables that could be used in quantitative studies. Finally, the variables considered in the articles obtained have been collected in a disparate manner making comparison difficult.

This study contributes to the advancement of scientific knowledge with knowledge of the actual profile of users of emergency contraception in Spain. This will allow us to program affective-sexual health strategies adjusted to the reality, at all levels: preventive, promotion, health education and health care; and reorient the accessibility of gynaecological services.

## Conclusion 

A major limitation found when performing the systematic review is that there are few studies published in the literature on emergency contraception in Spain and almost all of them are descriptive. The lack of homogeneity and comprehensiveness of the variables gathered only allows for a limited view of the profile of EC users and these individuals need to be studied in greater depth if educational interventions are to be designed within the framework of sexual health for potential users. In addition, studies are also urgently required comparing women who use EC with those who do not. Despite these drawbacks, using those variables that have data collected by the majority of articles, it can be observed that the profile of the EC user is a young, single woman who attends emergency services at the weekends, within 48 hours of unprotected coitus. Further study is required to understand the impact of free dispensation of EC, the profile of users and non-users, and their habits with respect to emergency contraception.
